# Inflammatory pseudotumor-like follicular dendritic cell sarcoma with first clinical manifestation of thrombocytopenia: A case report

**DOI:** 10.1097/MD.0000000000032528

**Published:** 2022-12-30

**Authors:** Dong Ni Leng, Kang-Jie Yu, Jing Wang

**Affiliations:** a Pathology department, Nanjing JunXie Hospital, Nanjing, China; b Translational Medicine Center, Nanjing JunXie Hospital, Nanjing, China.

**Keywords:** Barr virus, thrombocytopenia, Epstein, Inflammatory pseudotumor, like follicular dendritic cell sarcoma

## Abstract

Inflammatory pseudotumor-like follicular dendritic cell sarcoma (IPT-like FDCS) is often associated with Epstein–Barr (EB) virus infection. The tumor is commonly found in the spleen and liver, and it has been reported in the literature that it can be associated with paraneoplastic pemphigus, myasthenia gravis, and other diseases. A case of IPT-like FDCS with clinical features of thrombocytopenia has not been reported.

**Patient concerns:**

A 59-year-old male patient visited our hospital in September 2020 due to bleeding gums and epistaxis.

**Diagnosis:**

Splenic lymphoma with marked thrombocytopenia was initially diagnosed. The patient underwent pathological examination after splenectomy. Microscopic examination showed spindle-shaped or oval cells arranged in loose bundles, a large number of lymphocytes and plasma cells infiltrating the interstitium, and fibrin-like changes in the blood vessel wall. Immunohistochemical detection of tumor cells was positive for CD21, CD35, and Epstein–Barr virus in situ hybridization, and the patient was diagnosed with IPT-like FDCS.

**Interventions:**

The patient underwent a splenectomy. The patient received platelet-raising therapy postoperatively.

**Outcomes:**

No tumor recurrence or metastasis was found during the 17-month follow-up period, and the platelet count returned to normal.

**Conclusion:**

IPT-like FDCS is an uncommon tumor, and its first presentation with marked thrombocytopenia is even rarer. The tumor was clinically and radiographically nonspecific. Definitive diagnosis relies on histopathological and immunohistochemical staining. IPT-like FDCS is biologically indolent and has a favorable prognosis.

## 1. Introduction

Follicular dendritic cell (FDC) sarcomas are often confused with other tumors and inflammatory processes. Diagnosis requires the use of broad-spectrum FDC markers (e.g., CD21, CD23, CD35, clusterin, CXCL13, and podoplanin), especially given that tumor antigen loss is common. They can be divided into 2 types: conventional FDC sarcoma and Inflammatory pseudotumor (IPT)-like FDC sarcoma. In addition to its unique histopathological and clinical features, IPT-like FDCs are characterized by tumor cells that are positive for Epstein-Barr virus, which is necessary for diagnosis.^[1,2]^ IPT-like FDC sarcoma is a rare solid tumor with a favorable prognosis that usually occurs in the spleen and can involve other anatomical sites, including the liver and pancreas. The tumor tends to develop in young and middle-aged adults and may present with a variety of symptoms, including fever, abdominal pain, sweating, fatigue, and more commonly, a solitary lesion of the spleen. This sarcoma is a neoplastic spindle cell lesion usually characterized by FDC differentiation. It has been reported that paraneoplastic pemphegus monkeys, myasthenia gravis, and other diseases can be combined, and more than 90% of the cases are associated with EB virus.^[2–6]^ The diagnosis of IPT-like FDC sarcoma is problematic because of the rarity of cases and lack of specific clinical and imaging features. Therefore, we report a case of IPT-like FDCS with significant thrombocytopenia as the clinical symptom. The patient underwent platelet-raising therapy after surgical resection of the tumor. No tumor recurrence or metastasis was found during the 17-month follow-up period, and the platelet count returned to normal.

## 2. Case report

**Figure 1.**
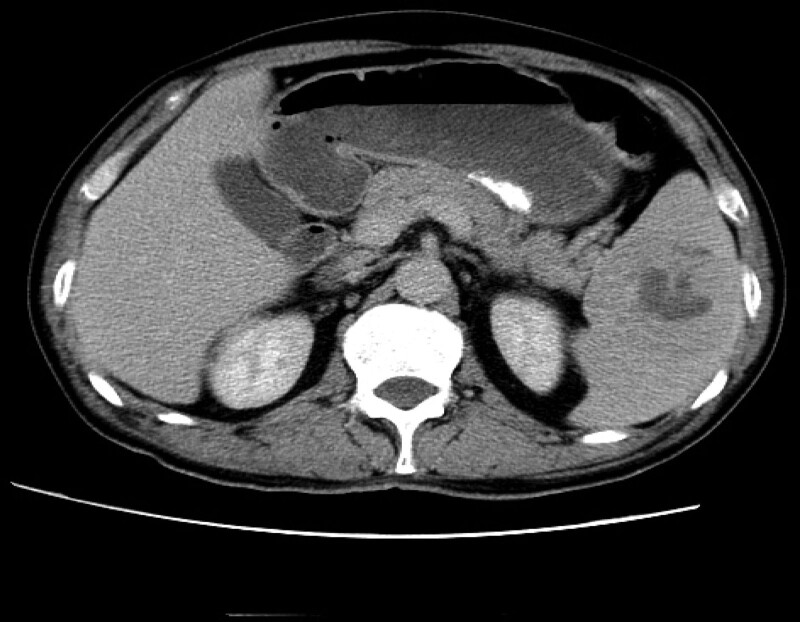
CT imaging before surgery. spleen mass and intrahepatic nodule, considering spleen tumor with intrahepatic metastasis.

A 59-year-old male patient presented with bleeding gums and epistaxis in January, 2020. The patient visited our hospital in September 2020. Blood routine showed total white blood cells 9.4 × 10^9^/L, monocytes 9.8%, hematocrit 0.279, hemoglobin 97 g/L, red blood cells 3.05 × 10^12^, platelets 12 × 10^9^/L, and platelet specific volume 0.01. Bone marrow smear showed that bone marrow hyperplasia was significantly active, and megakaryocytic hyperplasia was significantly active in maturation disorders. Abdominal and pelvic ultrasound showed spleen enlargement, solid mass in the spleen, lymphoma to be discharged, CT spleen with a slightly low-density mass (6 × 6.3 cm), increased FDG uptake, considering the malignant tumor, abdominal enhanced CT: spleen mass, and malignancy (Fig. 1). The patients underwent splenectomy and histopathological examination of the tumor specimens. Gross observation of pathological specimens: the mass is 9.5 × 5 × 3.5 cm in size, with clear boundary with surrounding tissues, gray-white solid on the cut surface, medium texture, and focal necrosis.

### 2.1. Morphological observation

**Figure 2.**
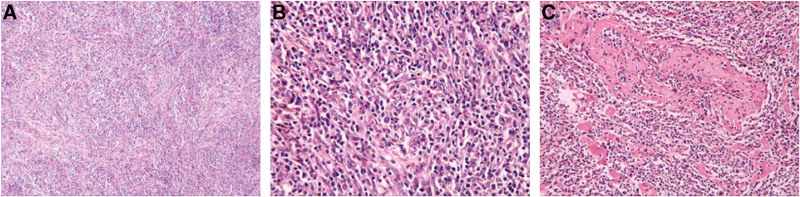
Routine pathological observation of tumor (a) Tumor cells are arranged in bundles. (b) Tumor cells are spindle-shaped or oval, with more lymphocytes and plasma cells infiltrated between cells. (c) Vascular wall fibrin-like changes.

**Figure 3.**
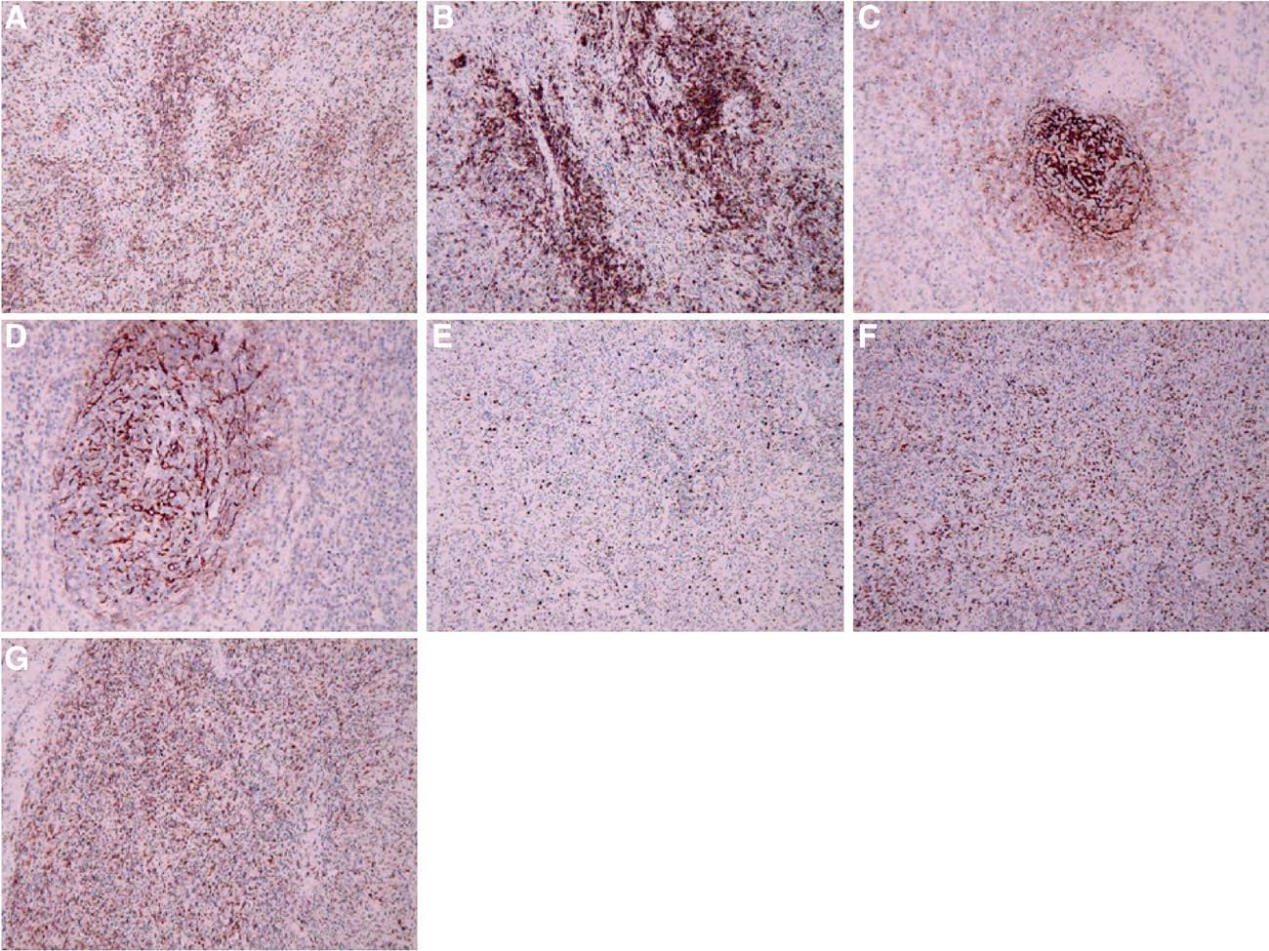
Immunohistochemical observation of tumor (magnification 100×, brown positive cells). (a) CD3+. (b) CD10+. (c) CD21+. (d) CD35+. (e) Ki-67 about 30%+. (f) Mum-1+.g. EBER+. EBER = Epstein–Barr virus in situ hybridization.

Tumor cells were arranged in loose bundles (Fig. 2a), with swirling structures visible locally, clear cell boundaries, spindle-shaped or oval, abundant cytoplasm, eosinophilic nuclei mostly long spindle-shaped, and nuclear staining plasma vesicular, with small and clear nucleoli and rare mitoses. Many lymphocytes and plasma cells infiltrated the tumor cells (Fig. 2b). Fibrin-like changes were observed in vessel walls (Fig. 2c). Local hemorrhage and sheet necrosis were observed in the tumor. Immunohistochemical results showed that CKpan-, CD20+, CD3 + (Fig. 3a), CD10 + (Fig. 3b), CD21 + (Fig. 3c), CD35 + (Fig. 3d), Bcl-2, Bcl-6, Ki-67 (+30%) (Fig. 3e), Mum-1 + (Fig. 3f), CD138++, κ+, λ+, CD43++, IgD-, and CD56 were scattered with a little +. Epstein–Barr virus in situ hybridization revealed a large number of Epstein–Barr virus-infected cells (Fig. 3g). PCR: B line gene rearrangement detection: IGH monoclonal band detection (−); IGK monoclonal band detection (−). The final pathological diagnosis was an IPT-like FDC. Their biological behavior is relatively lazy. Platelet-raising therapy was administered after the splenectomy. After 17 months of follow-up, no tumor recurrence or metastasis was observed, and the platelet count returned to normal.

## 3. Discussion

The clinical manifestation is that the pathogenic mechanism of significant platelet reduction is very complex. Pathological changes in the spleen lead to excessive platelet retention, resulting in peripheral blood thrombocytopenia.

IPT-like FDCS is an extremely rare low-grade malignant tumor. To date, only 48 cases have been reported, with a male-to-female ratio of 1:1.5. Clinical manifestations include abdominal pain, bloating, abdominal mass, weight loss, fever, fatigue, and anorexia, but most cases have no obvious symptoms.^[7]^ This case was reported for the first time, with thrombocytopenia as the first symptom. The patient had no other symptoms, except for bleeding gums or epistaxis.

The pathogenesis of IPT-like FDCS is associated with EB virus infection. It has also been documented that cases of IPT are associated with surgical or traumatic inflammation, autoimmune diseases, and infections, such as Castleman disease and pallidococcus,^[5,8–10]^ although other pathogenic mechanisms remain unclear. This case was positive for EBV and was accompanied by significant thrombocytopenia, suggesting that its pathogenesis was related to EB virus infection and pathological changes in the spleen. Splenic tumor infiltration leads to splenic platelet retention, resulting in marked peripheral thrombocytopenia.

IPT-like FDCS consists of neoplastic proliferative spindle cells with a large infiltration of lymphoplasmacytic cells. The borders between the cells are ill-defined and contain oval or elongated nuclei, vesicular or granular fine chromatin, and prominent nucleoli. Vascular fibrinoid changes were observed in this case, and EBV-induced cytokines and monokines are known to contribute to vascular proliferation, inflammation, and vascular injury,^[5]^ which may explain the vascular changes observed in our case.

Immunohistochemistry is crucial for diagnosing IPT-like FDCS. CD21 and CD35 are the preferred markers and support for the diagnosis of IPT-like FDCS. In this case, there were many plasma cells in the interstitium, CD138++, κ, and λ light chains were all positive, and CD43++, IgD-, and B-lineage gene rearrangement IGH and IGK monoclonal bands were all negative, so B-lineage lymphoma was excluded. Approximately 30% of Ki-67 is positive, indicating that the tumor is low-grade malignant; therefore, regular review and close attention are required.

In terms of treatment, surgical resection is usually the treatment of choice for patients with locally diseased tumors. The biological behavior of IPT-like FDCS is indolent and patients can survive for a long time after surgery. Long-term survival even after relapse.^[11]^ Platelet-raising therapy was administered after splenectomy. After 17 months of follow-up, no tumor recurrence or metastasis was observed, and the platelet count returned to normal.

## 4. Conclusion

An IPT-like FDCS is rare. In this report, cases of marked thrombocytopenia caused by pathological changes in the spleen caused by the tumor are even rarer. The clinical and imaging manifestations of this tumor are nonspecific, and definitive diagnosis depends on histopathology and immunohistochemical staining. Its biological behavior is indolent, and the prognosis is good after surgery and symptomatic treatment.

## Acknowledgments

The authors would like to thank the patient and her family for providing informed consent for publication.

## Author contributions

Dong-Li Leng conceptualized the study and drafted the manuscript; Kang-Jie Yu collected and analyzed the patient data; Jing Wang supervised the manuscript preparation. All authors have read and approved the final version of the manuscript.

**Data curation:** Kang-Jie Yu.

**Investigation:** Dong Ni Leng.

**Methodology:** Dong Ni Leng, Kang-Jie Yu.

**Project administration:** Dong Ni Leng.

**Writing—review and editing:** Jing Wang.

## References

[1] Facchetti F Simbeni M Lorenzi L. Follicular dendritic cell sarcoma. Pathologica. 2021;113:316–29. 34837090 10.32074/1591-951X-331 PMC8720404

[2] Cheuk W Chan JK Shek TW. Inflammatory pseudotumor-like follicular dendritic cell tumor: a distinctive low-grade malignant intra-abdominal neoplasm with consistent Epstein-Barr virus association. Am J Surg Pathol. 2001;25:721–31. 11395549 10.1097/00000478-200106000-00003

[3] Morales-Vargas B Deeb K Peker D. Clinicopathologic and molecular analysis of inflammatory pseudotumor-like follicular/fibroblastic dendritic cell sarcoma: a case report and review of literature. Turk Patoloji Derg. 2021;37:266–72. 34514557 10.5146/tjpath.2021.01523 PMC10510619

[4] Jiang L Hai-su T Dong C. Hepatic inflammatory pseudotumor-like follicular dendritic cell tumor with hepatic lymphoma history: a case report and literature review. Clin Case Rep. 2021;100:e27392. 10.1097/MD.0000000000027392 PMC8483863 34596165

[5] Jia-Yi Z Fang-Fei Z Qing-Wen L. Intra-abdominal inflammatory pseudotumor-like follicular dendritic cell sarcoma associated with paraneoplastic pemphigus: a case report and review of the literature. World J Clin Cases. 2020;8:3097–107. 32775392 10.12998/wjcc.v8.i14.3097 PMC7385594

[6] Xiyan W Huiyuan L Renchi Y. Immune imbalance in immune thrombocytopenia. Chin J Cell Biol. 2022;44:78–86.

[7] Bi-Xi Z Zhi-Hong C Liu Y. Inflammatory pseudotumor-like follicular dendritic cell sarcoma: a brief report of two cases. World J Gastrointest Oncol. 2019;11:1231–9. 31908727 10.4251/wjgo.v11.i12.1231 PMC6937438

[8] Madison M Stump MS Luyimbazi DT. Pancreatic inflammatory pseudotumor-like follicular dendritic cell tumor. Case Rep Pathol. 2019;2019:2648123 31885993 10.1155/2019/2648123 PMC6915151

[9] Tregnago AC Morbeck DL Costa FD. Inflammatory pseudotumor-like follicular dendritic cell tumor: an underdiagnosed neoplasia. Appl Cancer Res. 2017;37:45.

[10] Shuangshuang D Jinli G. Inflammatory pseudotumor-like follicular dendritic sarcoma: a rare presentation of a hepatic mass. Int J Clin Exp Pathol. 2019;12:3149–55. 31934158 PMC6949706

[11] Huayu H Xue Q Fengwei T. A rare case of primary pulmonary inflammatory pseudotumor-like follicular dendritic cell sarcoma successfully treated by lobectomy. Ann Transl Med. 2021;9:77. 33553370 10.21037/atm-20-4965 PMC7859798

